# Drug-Free Approach To Study the Unusual Cell Cycle of* Giardia intestinalis*

**DOI:** 10.1128/mSphere.00384-16

**Published:** 2017-09-20

**Authors:** Kathleen Horlock-Roberts, Chase Reaume, Guillem Dayer, Christine Ouellet, Nicholas Cook, Janet Yee

**Affiliations:** Department of Biology, Biochemistry and Molecular Biology Program, Trent University, Peterborough, Ontario, Canada; University at Buffalo

**Keywords:** polo-like kinase, RT-qPCR, actin-related protein, cyclins, geNorm, gene expression, histones, median body, minichromosome maintenance protein, proliferating cell nuclear antigen, thymidine kinase

## Abstract

*Giardia intestinalis* is a protozoan parasite that causes giardiasis, a form of severe and infectious diarrhea. Despite the importance of the cell cycle in the control of proliferation and differentiation during a giardia infection, it has been difficult to study this process due to the absence of a synchronization procedure that would not induce cellular damage resulting in artifacts. We utilized counterflow centrifugal elutriation (CCE), a size-based separation technique, to successfully obtain fractions of giardia cultures enriched in G_1_, S, and G_2_. Unlike drug-induced synchronization of giardia cultures, CCE did not induce double-stranded DNA damage or endoreplication. We observed increases in the appearance and size of the median body in the cells from elutriation fractions corresponding to the progression of the cell cycle from early G_1_ to late G_2_. Consequently, CCE could be used to examine the dynamics of the median body and other structures and organelles in the giardia cell cycle. For the cell cycle gene expression studies, the actin-related gene was identified by the program geNorm as the most suitable normalizer for reverse transcription-quantitative PCR (RT-qPCR) analysis of the CCE samples. Ten of 11 suspected cell cycle-regulated genes in the CCE fractions have expression profiles in giardia that resemble those of higher eukaryotes. However, the RNA levels of these genes during the cell cycle differ less than 4-fold to 5-fold, which might indicate that large changes in gene expression are not required by giardia to regulate the cell cycle.

**IMPORTANCE** Giardias are among the most commonly reported intestinal protozoa in the world, with infections seen in humans and over 40 species of animals. The life cycle of giardia alternates between the motile trophozoite and the infectious cyst. The regulation of the cell cycle controls the proliferation of giardia trophozoites during an active infection and contains the restriction point for the differentiation of trophozoite to cyst. Here, we developed counterflow centrifugal elutriation as a drug-free method to obtain fractions of giardia cultures enriched in cells from the G_1_, S, and G_2_ stages of the cell cycle. Analysis of these fractions showed that the cells do not show side effects associated with the drugs used for synchronization of giardia cultures. Therefore, counterflow centrifugal elutriation would advance studies on key regulatory events during the giardia cell cycle and identify potential drug targets to block giardia proliferation and transmission.

## Abstract (precis)

Giardias are among the most commonly reported intestinal protozoa in the world, with infections seen in humans and over 40 species of animals. The life cycle of giardia alternates between the motile trophozoite and the infectious cyst. The regulation of the cell cycle controls the proliferation of giardia trophozoites during an active infection and contains the restriction point for the differentiation of trophozoite to cyst. Here, we developed counterflow centrifugal elutriation as a drug-free method to obtain fractions of giardia cultures enriched in cells from the G_1_, S, and G_2_ stages of the cell cycle. Analysis of these fractions showed that the cells do not show side effects associated with the drugs used for synchronization of giardia cultures. Therefore, counterflow centrifugal elutriation would advance studies on key regulatory events during the giardia cell cycle and identify potential drug targets to block giardia proliferation and transmission.

## INTRODUCTION

*Giardia intestinalis*, a protist found in freshwater lakes and streams worldwide, is the causative agent of giardiasis, or “beaver fever,” a condition characterized by diarrhea, nausea, and abdominal cramping (1). It is also commonly associated with malnutrition and growth retardation in developing countries, in which the incidence of the disease is especially high (2). An estimated 2.8 × 10^8^ giardiasis infections occur in humans each year (3), making giardia one of the leading protozoan causes of intestinal diarrhea in the world. In addition to humans, giardia also infects a wide variety of other mammalian species, including livestock, pets, and wildlife (4, 5).

The giardia life cycle is characterized by two forms: a resistant, infectious cyst and a flagellated, pathogenic trophozoite. Ingestion of cysts via ingestion of contaminated water or food by a susceptible host initiates the infection. Passage of cysts through the acidic environment of the stomach triggers excystation and the release of trophozoites, which employ a ventral adhesive disk to attach to the lining of the upper intestine of the host (6). Trophozoites undergo multiple rounds of division by binary fission, enabling giardiasis infection from as few as 10 cysts (7). Movement of trophozoites to the lower intestine triggers encystation, where trophozoites differentiate to cysts that are excreted via the feces (8). Cysts serve as the source of future infection and are capable of surviving in lakes and rivers for 1 to 3 months (8).

Each giardia trophozoite contains two identical nuclei that are transcriptionally active and contain equivalent amounts of DNA (9, 10). Flow cytometry (FC) analysis results indicated that during normal vegetative growth, the ploidy of trophozoites alternates between tetraploid (2 × 2N per nucleus) and octoploid (2 × 4N per nucleus), which correspond to the G_1_ and G_2_ phases of the cell cycle, respectively (11). Unlike those of most eukaryotic species, the giardia trophozoite cell cycle features a predominant G_2_ phase, which also contains a restriction point for the differentiation of the trophozoites to infectious cysts (12). During encystation, the two 4N nuclei divide and DNA replication occurs without an accompanying cellular division, producing cysts with four nuclei and a total DNA ploidy of 16N (11).

The regulation of the cell cycle in giardia is integral to the control of the proliferation of trophozoites during an infection and their transition to infectious cysts. Furthermore, the giardia cell cycle has several unusual and unexpected features. For example, the giardia is able to continue to grow in size when DNA synthesis is blocked (13, 14) and is able to enter mitosis with double-stranded DNA breaks (15) or with defective spindles (16). These observations suggest that the giardia is able to override or has defective cell cycle checkpoints that are essential in other eukaryotic cells. To facilitate the analysis of gene expression changes occurring during this unusual cell cycle, giardia cultures must be manipulated such that samples enriched in each cell cycle phase can be collected. Many different techniques have been developed for the purpose of synchronizing the cell cycle of eukaryotes, with the majority of methods representing either whole-culture synchronization or physical separation techniques (17). Ideally, these methods should not introduce secondary effects or perturbations in cells. The treatment of trophozoite cultures with 5 μg/ml aphidicolin for 6 h (12) is one of the synchronization methods currently used for the giardia cell cycle. Such treatment results in the arrest of ~80% of cells in early S phase, which then progress synchronously through the cell cycle upon reincubation in aphidicolin-free media. Another giardia cell cycle synchronization protocol involves an initial incubation with 100 nM nocodazole for 2 h followed by 6 μM aphidicolin for 6 h, which results in the arrest of cells in G_1_/S (18). However, aphidicolin induces double-stranded DNA breaks (15) and nocodazole induces endoreplication (13, 14) as side effects in the treated giardia cells. Fluorouracil, hydroxyurea, colchicine, and demecolcine treatments and nutrient deprivation were also tested as agents for giardia synchronization but resulted in no effect on the giardia cell cycle, nonspecific blocks, or irreversible effects (14). Although it is possible to use flow cytometry to analyze giardia trophozoites and to isolate encysting giardia and cysts (11), it is currently not possible to use this technique to isolate nonencysting vegetative giardia trophozoites (unpublished data). The likely explanation is that giardia trophozoites are lysed during the sorting process.

For this report, we explored the application of counterflow centrifugal elutriation (CCE), a drug-free method, to obtain sufficient numbers of giardia trophozoites at stages from G_1_ and S to G_2_ for further experimental analysis. An elutriation experiment involves the operation of a specially designed elutriation chamber and rotor housed within a centrifuge. Tubing connected to a peristaltic pump allows the passage of a buffer through the elutriation system at a controlled speed. The flow of buffer delivered by the pump transports injected asynchronous cells into the elutriation chamber, where cells are prevented from exiting the chamber by the centrifugal force opposing their movement (19). The distribution of cells within the chamber is dependent on cell size, as small, slowly sedimenting cells migrate farthest and settle closest to the chamber outlet, while large cells remain closest to the chamber’s inlet (19). An increase in the flow rate causes all cells to move toward the outlet tube, with the smallest cells being eluted first for collection. Incremental increases in the flow rate from this point allow increasingly larger cells to be collected in sequential fractions. Since the cells used in CCE are not subjected to drugs, this technique allows the collection of samples that should more closely approximate the state of cells at the different cell cycle stages. Moreover, the ability to load a large number of cells into the CCE system (10^7^ to 10^9^ cells for the standard elutriation chamber) and short separation times mean that cells representative of all cell cycle phases can be collected rapidly in one single experiment (17). For these reasons, CCE has been used to produce samples for analysis of the cell cycles of several protozoa, including *Tetrahymena* (20), the dinoflagellate *Amphidinium carteri* (21), *Paramecium tetraurelia* (22), and *Trypanosoma brucei* (23).

The determination of gene expression profiles from the comparison of RNA levels corresponding to genes of interest requires the normalization of data to minimize unwanted variation due to nonbiological effects. In RT-qPCR assays, the most common normalization method is to use a reference gene that has a constant RNA level under the different biological conditions or samples evaluated in the study to correct for technical variation. The selection of the most appropriate reference gene for an experiment requires careful consideration, as a gene that performs well as a reference for the study of one set of biological conditions may have different RNA levels under a different set of conditions. We evaluated six housekeeping genes as potential normalizers for the RT-qPCR analysis of the CCE fractions by the geNorm program.

## RESULTS

Although the majority of trophozoites in an asynchronous giardia culture are in the G_2_ stage of the cell cycle (11), we asked if there is a particular growth phase in the culture that contained the highest fraction of G_1_-phase and S-phase cells that we could use for CCE fractionation. Consequently, a culture of giardia trophozoites was grown at 37°C for 60 h, and samples of the culture at different time points were subjected to cell enumeration to determine cell densities and flow cytometry (FC) to determine the distributions of cells among the different cell cycle stages. Although the fraction of G_1_/S cells remained low relative to the fraction of G_2_ cells throughout the growth period, the highest proportion of G_1_/S cells was found in the culture at early to mid-log phase, which corresponds to a density of 3 × 10^5^ to 6 × 10^5^ cells/ml (data not shown).

**FIG 1:**
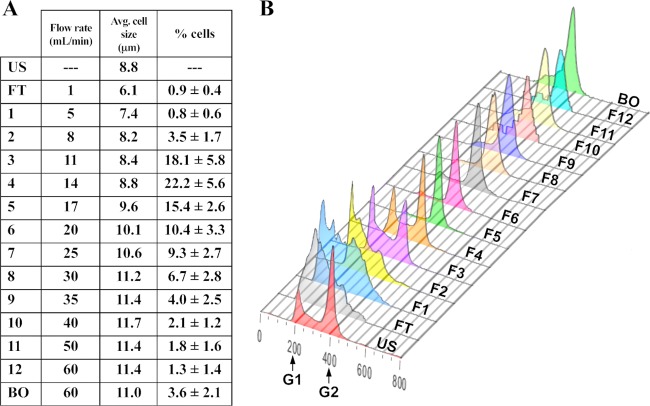
Cell cycle and cell size analysis of elutriation fractions. (A) The flow rate determined for each CCE fraction is listed in column 2. The average cell size in each sample was determined by analysis performed on a ViCell XR cell viability analyzer (Beckman-Coulter). The percentage of cells recovered in each fraction was determined by the number of cells in the fraction divided by the sum of the cells recovered in all fractions. The average (Avg.) percentage and standard deviation were calculated from five independent elutriation experiments. (B) Flow cytometry analysis of a representative set of elutriation samples. The data corresponding to the *x* axis of the histogram represent relative SYTOX green fluorescence levels per cell, while the *y* axis data represent cell number, with each sample being normalized to the highest peak. The assignments of the peak of G_1_ cells centered at 200 and the peak of G_2_ cells centered at 400 on the *x* axis were based on calibrations performed previously (11, 14). US, unsorted asynchronous control sample; FT, flowthrough fraction; BO, blowout fraction.

We tested different combinations of centrifugal force and pump flow rate to load the giardia trophozoites into the CCE system. A centrifugal force level of 550 × *g* and an initial flow rate of 1 ml/min allowed the injected trophozoites to be retained in the CCE system, with less than 1% of the input cells lost in the flowthrough (FT) fraction (Fig. 1A). Fractions were collected at increasing increments of the flow rate, while the centrifugal force was held constant at 550 × *g*. A final fraction was collected at 55 ml/min with the centrifuge rotor stopped (0 rpm) to eluted all remaining cells in the system; this is referred to as the blowout fraction (BO).

Analysis of the CCE samples indicated that the majority of cells was eluted in fractions 3 to 7, which corresponded to flow rates of 11 to 25 ml/min, and that the average cell size in each sample increased slightly from FT until F10 (Fig. 1A). FC analysis to determine the distribution of cells within the cell cycle within each CCE fraction showed that the initial fractions (FT, F1, and F2) contained predominantly G_1_/S cells (Fig. 1B), with fraction 2 containing the highest number of cells among the three samples (Fig. 1A). Fractions 5 and higher showed a high proportion of G_2_ cells (Fig. 1B), with fraction 5 containing up to 90% of G_2_ cells in the total cell population (Fig. 1A).

To test the viability of the cells recovered in the elutriation fractions, we used fraction 7, which was enriched in G_2_ cells, to inoculate multiple 1.5-ml cultures in parallel to complete a growth curve analysis. We took cell counts in triplicate for 10 time points over a 46-h period. The doubling time was determined to be approximately 8.5 h, which is in the range of the doubling times of 7 to 9 h that we observed with unsorted giardia cultures in our laboratory.

**FIG 2:**
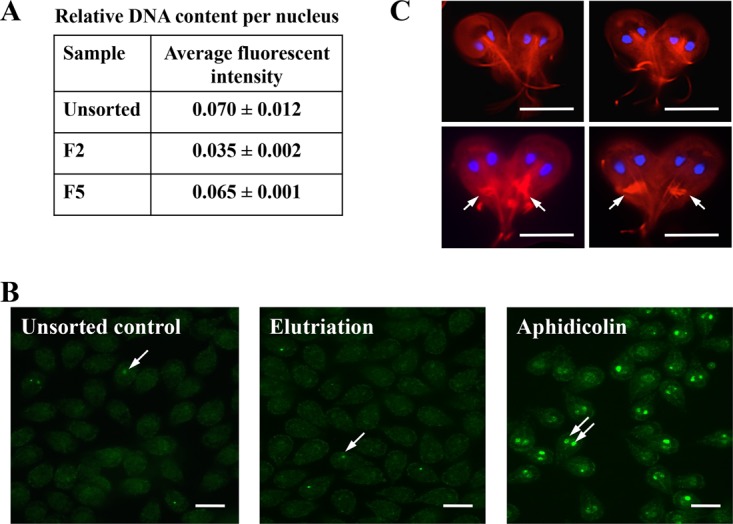
Analysis of DNA content, DNA damage, and cell division in elutriation fractions. (A) The average intensity of each DAPI-stained nucleus was measured in an unsorted and asynchronous giardia sample and elutriation fractions 2 and 5 using the CellProfiler program. The average fluorescent intensity/nucleus was calculated from the values obtained from 100 cells in each sample. (B) Immunofluorescence detection of phosphorylated histone H2AX in giardia trophozoites. A small percentage of cells in the asynchronous control giardia culture contained a positive signal that was usually localized in only one of the two nuclei per cell (single arrow). Similarly, an elutriation fraction also contained few cells with a positive signal in only one nucleus per cell (single arrow). Trophozoites incubated with 5 μg/ml aphidicolin for 6 h showed a positive signal in approximately 50% of cells, usually in both nuclei (double arrows). Bar: 10 μm. (C) Heart-shaped cells in later elutriation fractions. Nuclei were stained with DAPI (blue), and tubulin structures, including the median bodies (indicated by arrows), were stained with the TAT-1 monoclonal antibody. Bar: 7.5 μm.

The CellProfiler program (24) was used to measure the fluorescence level of the nuclei in the trophozoites recovered in fractions 2 and 5 after DAPI (4′,6-diamidino-2-phenylindole) staining as an estimation of the DNA content of these cells. The average fluorescent intensity of each nucleus was 0.035 ± 0.002 for fraction 2 and 0.065 ± 0.0016 for fraction 5, which resulted in a ratio of 1:1.9 (Fig. 2A).

Samples of giardia trophozoites were also examined by immunofluorescent microscopy with an antibody for phosphorylated histone H2AX (γH2AX), which is a marker of double-stranded DNA damage. In samples from an unsorted giardia culture (not subjected to CCE), γH2AX was observed in at least one nucleus in 8% of trophozoites and in both nuclei in 1% to 2% of trophozoites (Fig. 2B). Among the CCE fractions, the proportion of cells with γH2AX in at least one nucleus ranged from 3% to 11%, and the proportion of cells with γH2AX in both nuclei ranged from 0.5% to 5% (Fig. 2B). In contrast, giardia cultures treated with 5 μg/ml aphidicolin for 6 h had up to 50% of cells with γH2AX in at least one nucleus and 30% of cells with γH2AX in both nuclei (Fig. 2B).

**FIG 3:**
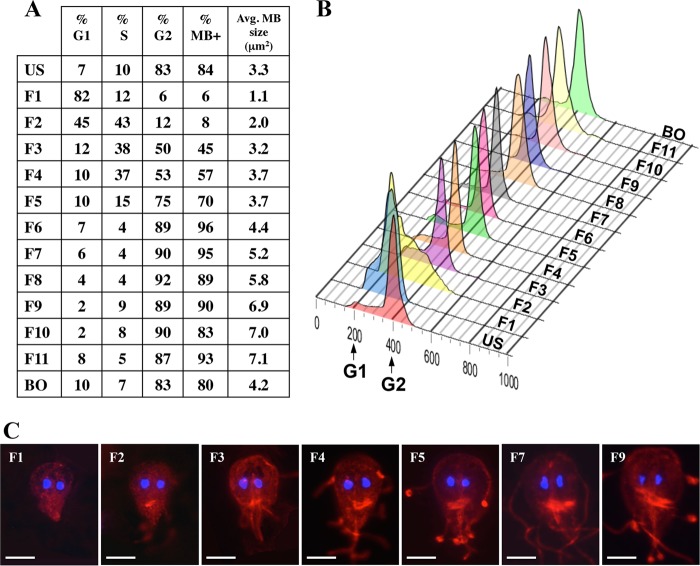
Analysis of median body in elutriation fractions. (A) The cell cycle distribution of cells in each elutriation fraction was determined by analyzing the flow cytometry data with FlowJo software. Median bodies were stained by the TAT-1 antibody and visualized under epifluorescence microscopy. The percentages of cells containing median bodies were determined in the elutriation fractions. The relative size (in square micrometers) of each median body was determined by analysis of microscopy images with ImageJ software as described in Materials and Methods. The average size of the median body in each fraction is shown in the last column. (B) Flow cytometry analysis of the set of elutriation samples used for the analysis of the median body. The *x* axis of the histogram represents relative SYTOX green fluorescence levels per cell, while the *y* axis represents the cell number, with each sample being normalized to the highest peak. US, unsorted asynchronous control sample; FT, flowthrough fraction; BO, blowout fraction. (C) Representative cells from each of several fractions are shown to illustrate the appearance and increase in the size of the median body as the collection of CCE fractions increased. The fraction number is indicated in the upper left corner of each image. The median bodies are the bright red fluorescent structures beneath the two DAPI-stained nuclei (blue) in each cell. Bar: 4.5 μm.

Microscopic examination of the CCE fractions showed that fractions 8 to 10 contained a 10-fold to 20-fold enrichment of heart-shaped trophozoites (Fig. 2C), which were described in previous publications as representative of giardia in the process of cytokinesis (25–28). This result, along with the low (1.3%) mitotic index in giardia (26) and interest in the study of mitosis due to several conflicting reports published about this mechanism in giardia, prompted us to determine whether it is possible to identify an elutriation fraction enriched in cells containing mitotic spindles. Despite several attempts using the TAT-1 monoclonal antibody against α-tubulin (29), we could not identify such a fraction. However, we noticed that the appearance and size of the median body that is also stained by the TAT-1 changed among the cells in these fractions (Fig. 3). The median body is a structure unique to giardia. The function of the median body is unknown, but it is thought to store components of the ventral disc (30). There is evidence that the median body is very small or absent in G_1_, grows in size during S and G_2_, and then disappears during mitosis (15, 31). In the elutriation results shown in Fig. 3A, the median body was found in more than 80% of the cells in the unsorted asynchronous (US) faction. This proportion dropped to 6% in fraction 1 but steadily increased to more than 90% in fraction 6. Moreover, the average size of the median body was smallest in fraction 1 but steadily increased until fraction 11. Cells representative of several elutriation fractions are shown in Fig. 3C to illustrate the appearance and increase in the size of the median body among these fractions.

**FIG 4:**
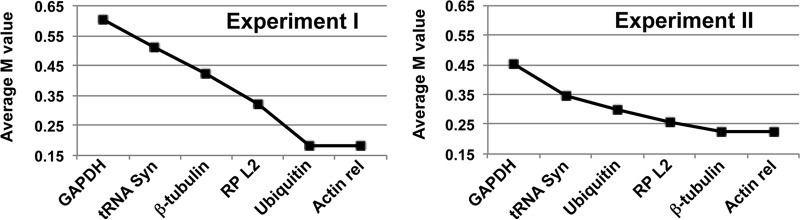
Average expression stability of six candidate normalizer genes determined by geNorm. The expression stabilities for the giardia genes encoding the actin-related protein (Actin rel), β-tubulin, GAPDH, glycyl tRNA synthetase (tRNA Syn), ribosomal protein L2, and ubiquitin were determined by the geNorm program for two independent elutriation experiments. Genes are plotted in the graphs from least to most stable from left to right.

We next examined the expression profiles of several genes by measuring their corresponding mRNA levels in the CCE fractions by RT-qPCR. However, we first needed to select an appropriate gene for normalization of the mRNA levels during the giardia cell cycle. The geNorm program (32) was used to test six giardia genes encoding the following products as potential normalizers: actin-related protein (GL50803_15113), β-tubulin (GL50803_101291, GL50803__136020, and GL50803__136020), GAPDH (glyceraldehyde-3-phosphate dehydrogenase) (GL50803_6687), glycyl tRNA synthetase (GL50803_9011), ribosomal protein L2 (GL50803_16086), and ubiquitin (GL50803_7349). The geNorm program calculates expression stability measurements (M values) for all the genes, with the most stable genes (i.e., those most suitable for use as normalizers) possessing the lowest M values. Analysis of the mRNA from fractions from two independent elutriation experiments showed that the giardia actin-related gene had the lowest M value (Fig. 4).

The expression levels of 11 genes were measured among the CCE fractions by RT-qPCR using the actin-related gene as the normalizer. These analyses were restricted to fractions 2 to 10 because the most consistent yields of RNA were obtained from these fractions. On the basis of the FC results, we classified fractions 2 and 3 as G_1_/S fractions, as these samples contained an enrichment of G_1_-phase and S-phase cells compared to the asynchronous unsorted control, while the remaining fractions were classified as G_2_. Fraction 5 was chosen as the calibrator to determine the relative expression levels for the other CCE fractions, as this is the most highly G_2_-enriched fraction, and high percentages of cells were consistently recovered from this fraction in our elutriation experiments.

**FIG 5:**
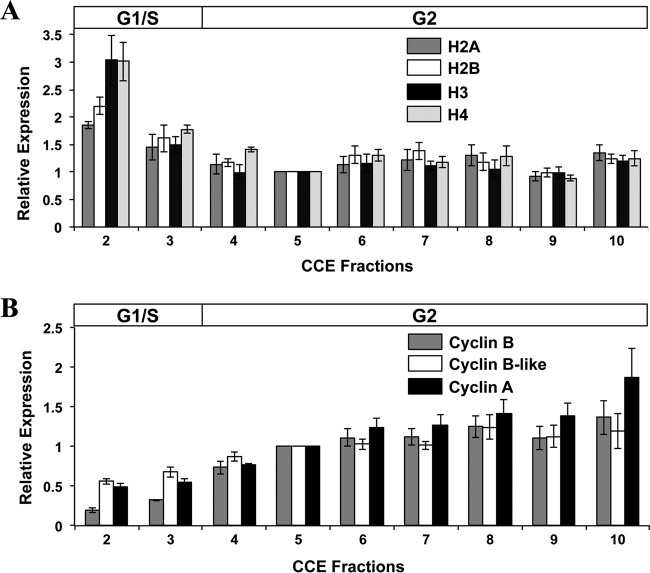
Transcript levels for core histone genes and cyclin genes in elutriation fractions. (A) Relative mRNA levels of the four core giardia histone genes: H2A, H2B, H3, and H4. (B) Relative mRNA transcript levels of the giardia cyclin B, B-like, and A genes. The average expression levels of these genes were calculated from four separate RT-qPCR analyses of fractions collected in two independent elutriation experiments. The RT-qPCR data were normalized to the actin-related gene, and the expression levels are shown relative to those determined for fraction 5 as the calibrator. The G_1_/S and G_2_ bars above the graph denote the predominant cell type in the fractions according to FC analysis results.

**FIG 6:**
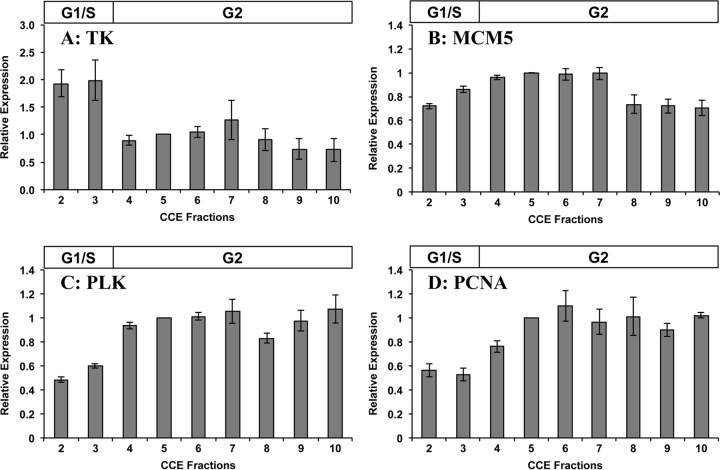
Expression levels of additional giardia genes in elutriation fractions. Data represent transcript levels of the giardia genes encoding (A) thymidine kinase (TK), (B) minichromosome maintenance 5 (MCM5), (C) polo-like kinase (PLK), and (D) proliferating cell nuclear antigen (PCNA). The average expression levels of these genes were calculated from four separate RT-qPCR analyses of fractions collected in two independent elutriation experiments. The RT-qPCR data were normalized to the actin-related gene, and the expression levels are shown relative to those determined for fraction 5 as the calibrator. The G_1_/S and G_2_ bars above the graph denote the predominant cell type in the fractions according to FC analysis results.

Our RT-qPCR data show that the mRNA levels of the four core histone genes of giardia all peaked in the G_1_/S-phase-enriched fractions at levels ~2-fold to 3-fold higher than in the G_2_ phase (Fig. 5A). In contrast, the transcript levels of three giardia cyclin genes were all highest in G_2_-enriched elutriation fractions (Fig. 5B). The cyclin B mRNA levels were 7-fold higher in the last G_2_ fraction (fraction 10) than in the most highly enriched G_1_/S fraction (fraction 2), while a 2-fold increase was also observed with a cyclin B-like gene and the cyclin A gene. The mRNA level for the giardia thymidine kinase (TK) gene was 2-fold higher in the G_1_/S fractions than in the remaining fractions (Fig. 6A), while the minichromosome maintenance 5 (MCM5) gene had a slight increase in transcript levels in G_2_ fractions 4 to 7 followed by a slight decrease in the subsequent fractions (Fig. 6B). The genes for polo-like kinase (PLK) and proliferating cell nuclear antigen (PCNA) had 2-fold higher mRNA levels in the G_2_ fractions than in the G_1_/S fractions (Fig. 6C and D).

## DISCUSSION

A feature of the progression of cells through the cell cycle is the accumulation of DNA, proteins, and other cellular components at each stage, contributing to an increase in cell size from the early G_1_ phase to the late G_2_/M phase (33). Cells in G_1_ phase must reach an adequate size before DNA synthesis can initiate (34). Consequently, cell size is a distinguishing feature of each individual cell cycle phase and is a quantity that increases from early G_1_ until the completion of cytokinesis. Counterflow centrifugal elutriation (CCE) generates fractions from a mixed cell population based on cell size. The goal of this study was to develop the counterflow centrifugal elutriation technique for the physical fractionation of asynchronous giardia trophozoite cultures to generate samples enriched in the different cell cycle phases. We first determined that trophozoite cultures grown to mid-exponential phase (~3 × 10^5^ to 6 × 10^5^ cells/ml) contained the highest proportions of G_1_-phase and S-phase cells. Next, we determined the centrifugal force, the initial flow rate, and the flow rate increments to apply to these cultures for successful fractionation. We established one set of elutriation parameters that resulted in sequential fractions containing giardia trophozoites with increasing average cell sizes (Fig. 1A). FC analysis showed that the fractions collected at increasing flow rates also corresponded to the enrichment of cells progressing from the G_1_ phase to the S phase and G_2_ phase of the cell cycle (Fig. 1B).

Cell Profiler analysis was performed on the DAPI-stained samples from fractions 2 and 5, which were the fractions identified by FC analysis to contain high proportions of G_1_/S cells and G_2_ cells, respectively (Fig. 2A). The 1:2 intensity ratio of the DAPI fluorescence of the cell nuclei in these samples suggests that fraction 2 contained mainly G_1_ cells and faction 5 contained mainly G_2_ cells. The purity of the G_1_ cells in fraction 2 and of the G_2_ cells in fraction 5 is indicated by the relatively low standard deviation associated with the average fluorescence intensities in these samples compared with the much higher standard deviation associated with the average fluorescence value for the unsorted cells from an asynchronous giardia culture (Fig. 2A). Furthermore, we did not detect trophozoites with levels of DNA content significantly greater than expected for G_2_ cells in fractions 5 to 10 (data not shown), which suggests that the elutriation process did not induce endoreplication. We also showed that the elutriation process did not induce double-stranded DNA breaks as indicated by the low detection level of phosphorylated histone H2A in cells from the CCE fractions (Fig. 2B).

Microscopic examination of the CCE fractions also showed that fractions 8 to 10 contained a 10-fold to 20-fold increase in the level of heart-shaped cells compared to an unsorted culture (Fig. 2C). Initially, we thought that all these heart-shaped cells were in the process of cytokinesis. However, recent studies utilizing live imaging of giardia trophozoites showed that heart-shaped cells that retain median bodies are arrested in cytokinesis rather than being actively dividing cells and that the transfer of giardia trophozoites out of growth medium into a buffered solution such as HEPES-buffered saline (HBS) allowed only a 5-min window for the cells to complete cytokinesis before they became stuck (35). Since we performed the elutriation in phosphate-buffered saline (PBS) and more than half of the heart-shaped cells shown in Fig. 2C contained median bodies, it is likely that these cells were arrested in cytokinesis and that the reason that they were enriched in the latter fractions is that they had an increased overall cell size. Our use of PBS or HBS as the elutriation buffer is also the reason that we were unable to enrich cells containing a mitotic spindle in our elutriation fractions. We have tried to use giardia growth medium (TYI-S-33) as the fluid for elutriation, but this resulted in poor recoveries of cells in the fractions. This was likely due to the increased adhesion of the cells to the tubing during the elutriation process and was possibly due to the presence of one or more of the medium components. The use of a modified giardia medium as the elutriation fluid would be a promising avenue to explore to obtain an enrichment of mitotic giardia via CCE.

We observed changes in the prevalences and the sizes of the median bodies among the cells in the elutriation fractions (Fig. 3). The median body is a structure unique to giardia and serves as a reservoir of tubulin and other cytoskeletal components (30, 36). The median body is thought to be a ready source of components to allow the quick assembly of a replicate ventral disk during mitosis such that both daughter cells would be able to use the ventral disc to attach to the host epithelial cells and prevent their removal by peristaltic flow in the intestinal tract (30, 36). The median body is very small or absent in G_1_ cells, grows during S and G_2_ cells, and then disappears during mitosis (15, 31). These observations, together with the observation that the median body disassembles during mitosis via live imaging of giardia cell division (35), support the idea of a role of the median body as a source of materials for building the ventral discs during mitosis. We observed that the prevalence of the median body increased among the cells from increasing elutriation fractions (Fig. 3A). In addition, we observed an increase in the average size of the median body as the elutriation fractions increased (Fig. 3A and C). These data support the suitability of the CCE method to study other giardia cellular structures and organelles during the cell cycle.

A highly sensitive technique for the determination of gene transcript levels within and between different biological samples is reverse transcription-quantitative PCR (RT-qPCR). However, RT-qPCR data must be normalized to control for differences in RNA quantity and quality and cDNA synthesis efficiency between samples. The most frequently used normalization strategy involves measuring the RNA of the gene of interest relative to the RNA of a reference gene with transcript levels that remain constant under the experimental or biological conditions being studied (37). Common reference genes include those encoding GAPDH (glyceraldehyde-3-phosphate dehydrogenase), α- and β-actin, tubulin, and 18S and 28S rRNAs (38). However, extensive evidence suggests that the transcript levels of these genes fluctuate in various cell types and under certain experimental conditions (38). One common method to evaluate candidate reference genes is to use the geNorm program, which gives a stability score (M value) based on pairwise comparison of the nonnormalized expression values of these genes across the experimental samples of interest (32). We used the geNorm program to determine M values for the giardia actin-related, β-tubulin, GAPDH, glycyl tRNA synthetase, ribosomal protein L2, and ubiquitin genes across fractions 2 to 10 collected from two independent CCE experiments. The geNorm results from both CCE experiments showed that the actin-related gene was the most stable and the GAPDH gene was the least stable among the samples analyzed in each experiment (Fig. 4).

We used RT-qPCR to examine the transcript levels of 11 genes during the giardia cell cycle with RNA from the CCE fractions and the actin-related gene as the normalizer. Each of the giardia genes selected is an ortholog of a gene in at least one other eukaryotic species that has been determined to exhibit cell cycle-regulated expression. Our results showed that 10 of these genes (the genes encoding four core histones, cyclin B, cyclin B-like, cyclin A, thymidine kinase, MCM5, and PLK) have cell cycle expression profiles that are similar to those in other eukaryotes. Only the giardia PCNA gene showed a cell cycle expression pattern that differed from the expected pattern.

The core histones (H2A, H2B, H3, and H4) are among the most highly conserved and extensively studied cell cycle-regulated genes in eukaryotes. Since one of their roles is to organize newly synthesized DNA into chromatin, the levels of the histone mRNAs increase in the G_1_ and early S phases in yeast, *Drosophila*, and mammalian cells (39–41). In a previous study, the mRNAs of the giardia histone H2A, H2B, and H4 genes were shown to be upregulated by 5-fold to 7-fold in G_1_/S-phase trophozoite cultures synchronized by aphidicolin (12). Our analysis of elutriation fractions of giardia trophozoite cultures also showed upregulation by the four core histone mRNAs in the G_1_ and S phases (Fig. 5A) but only by 2-fold to 3-fold. As aphidicolin blocks DNA synthesis by binding to DNA polymerase, cells that are released from this block may show a slightly higher increase in the expression of S-phase regulated genes as an initial response to the release from this drug.

Cyclins, originally discovered as proteins whose levels fluctuate during the cell division cycle of sea urchin eggs (42), represent a major class of regulators of the eukaryotic cell cycle. The mRNA and protein levels of cyclins change during the cell cycle, reaching peaks during the phase in which their activity is required. Generally, B-type cyclins associate with cyclin-dependent kinase 1 (CDK1; also known as cell division control 2, or CDC2), and the resulting cyclin B/CDK1 complexes serve as the maturation- or mitosis-promoting factors in eukaryotic cells (43). B-type cyclin proteins are degraded by the anaphase-promoting complex (APC) at the metaphase-anaphase transition to allow further progression through mitosis (44). Interestingly, the giardia genome does not appear to encode any components of the APC complex (45). Despite this, the protein level of cyclin B is low in giardia cells at anaphase and cytokinesis compared to the 4-fold to 5-fold increase in the levels of this protein in S and G_2_ cells (45). This result suggests that cyclin B is degraded late in mitosis in a process that is independent of the ubiquitin pathway (45). In other eukaryotes, the transcript level of cyclin B is upregulated by 4-fold to 7-fold in late G_2_/M phase (46–48). In giardia cultures synchronized by aphidicolin, there was a gradual increase in the mRNA level of cyclin B from the G_1_/S phase until it reached a 12-fold increase in the G_2_ phase (12). Our results also showed a gradual increase in the mRNA level of cyclin B that reached its peak in late G_2_ phase (fraction 10) at a level 7-fold higher than that seen with the G_1_/S phase (fraction 2) (Fig. 5B). This increase in the level of cyclin B mRNA more closely matches the 4-fold to 5-fold increase in the cyclin B protein level observed by Gourguechon et al. (45). We also examined the mRNA level of a cyclin B-like gene in giardia. The mRNA level of this gene also gradually increased starting from the G_1_ phase, but it was found to have increased by less than 2-fold at the G_2_ phase, and it remained relatively constant among the remainder of the CCE fractions (Fig. 5B). The expression profile of the cyclin B-like gene suggests that its protein has a role that is similar or complementary with respect to the function of cyclin B in the G_2_/M phase of the giardia cell cycle.

A-type cyclins are capable of associating with and activating two independent *cdk* genes to exert effects on both DNA synthesis and mitosis (49). During the G_1_/S transition, cyclin A associates with CDK2, and this complex localizes to DNA replication foci during the S phase (50). Furthermore, the elements of the cyclin A/CDK2 complex have possible roles in promoting DNA synthesis or in preventing extraneous replication (51). From the late S phase until its degradation in metaphase, cyclin A associates with CDK1 (49). Cyclin A also has important roles in the inactivation of WEE1, an event that is required for the subsequent activation of cyclin B/CDK1 complexes during entry into mitosis (52). Analysis of cyclin A2 knockdown HeLa cells indicates that this protein may have roles in cyclin B1 nuclear translocation, chromatin condensation, and nuclear envelope breakdown (53). However, the nuclear envelope in giardia does not completely disassemble during mitosis but remains semiopen (26, 54). Our results show that the mRNA level of the giardia cyclin A gradually increases starting from a G_1_-enriched sample (fraction 2) and reaches a peak in a late G_2_-enriched sample (fraction 10).

The thymidine kinase (TK) enzyme is a component of the pyrimidine deoxyribonucleotide salvage pathway that catalyzes the phosphorylation of thymidine to form dTMP (55). The expression and activity of the TK enzyme are closely associated with cell cycle progression, as their levels increase at the G_1_/S transition and remain elevated until they rapidly decrease during mitosis (56). Giardia appears to lack the ribonucleotide reductase pathway for *de novo* deoxyribonucleotide synthesis (57), instead relying on the salvaging of existing purines and pyrimidines. Thymidine kinase is one of the few deoxynucleoside kinases in the giardia genome (58) involved in the replenishment of the nucleotide pool during each cell cycle. In giardia, a putative E2F1-like protein was found to bind and transactivate the thymidine kinase promoter, indicating that this gene may be cell cycle regulated at the G_1_/S transition in this parasite (59). Indeed, our RT-qPCR analysis of the thymidine kinase mRNA levels in the elutriation samples demonstrated that this gene is upregulated in the fractions enriched in G_1_/S cells (Fig. 6A).

Minichromosome maintenance protein 2 (MCM2) to MCM7 assemble to form a heterohexameric ring structure at the origins of DNA replication (60). The helicase activity of the MCM ring separates the DNA strands so that DNA synthesis can begin. Generally, MCM gene transcript levels peak in early G_1_/S phase or late mitosis (61, 62). The giardia genome contains orthologs for all six core genes (encoding MCM2 to MCM7) (63). The analysis of our giardia elutriation samples showed a slight peak in MCM5 mRNA levels in the G_1_/S and early G_2_ phases (Fig. 6B), although this increase was small relative to its lowest level in the later CCE fractions (F8 to F10).

Polo-like kinase (PLK) was first identified in *Drosophila* larval neuroblasts, where mutations in the polo gene contributed to the formation of abnormal mitotic spindles and progression through mitosis (64). Phosphorylation and activation of CDC25 are major functions of PLK, which in turn phosphorylates and activates CDK1/cyclin B to promote mitotic entry (65). Additional roles for PLK have been shown in centrosome maturation and separation, mitotic spindle formation, removal of cohesin to promote sister chromatid separation, anaphase-promoting complex activation, and exit from mitosis and cytokinesis (66). Results of BLAST analysis of the giardia genome indicate the presence of a single PLK gene encoding a protein containing polo box domains (67). Our RT-qPCR analysis of the CCE fractions showed that the peak mRNA level of the giardia PLK occurred in the G_2_ phase (Fig. 6C), which is consistent with the mRNA expression patterns observed in other eukaryotic species (47, 68, 69).

Proliferating cell nuclear antigen (PCNA) forms a homotrimeric sliding clamp that tethers DNA polymerase to the DNA during replication (70). Notably, PCNA interacts with the CDC25 protein during the G_2_/M phase (71) and has been shown to interact with mitotic cyclins A and B and their corresponding *cdk* genes (72), suggesting possible roles for PCNA in mitosis. The general pattern of PCNA mRNA expression is an increase during the G_1_/S transition or S phase of the cell cycle (47, 73). In giardia, the transcript levels of the putative PCNA gene peaked in elutriation fractions enriched in G_2_ cells (Fig. 6D). The S-phase-dependent transcription of the human PCNA gene is influenced by members of the E2F family of transcription factors (74, 75). However, overexpression of a putative E2F1-like protein in giardia did not have any significant effect on PCNA gene expression (59). In *Toxoplasma gondii* tachyzoites, there are two distinct PCNA proteins that differ in their cell cycle-dependent cellular localization and ability to rescue PCNA mutants in yeast (76). Also, two distinct PCNA proteins in *Plasmodium falciparum* with different life cycle-dependent expression patterns have been previously identified (77). Although only one PCNA gene has been annotated in the giardia genome, it is possible that giardia may have a second PCNA gene that has not been identified due to its sequence divergence. In this scenario, the PCNA gene analyzed in this study may encode a protein that has a different role in the cell cycle, possibly with a function such as DNA repair in the G_2_/M phase. Alternatively, if the PCNA that we studied is the only ortholog in giardia, then this protein may have dual roles in the G_1_/S phase and in the G_2_/M phase as observed in human PCNA (71).

The cell cycle expression profiles for 10 of the 11 genes that we examined using the CCE fractions are similar to those for other eukaryotes and indicate that our enriched elutriation samples are representative of the different cell cycle phases. However, the changes in transcript levels during the giardia cell cycle were modest compared to those seen with the orthologous genes in model eukaryotic species (41, 48, 62, 78–80). On the other hand, our results are consistent with the changes in transcript levels observed during the cell cycles of other protozoan parasites. The most highly regulated cell cycle genes in *Trypanosoma brucei* procyclic cells showed only 4-fold to 5-fold changes in their mRNA levels (23). Similarly to our results, the mRNA levels of the *Trypanosoma cruzi* core histone genes peaked in the S phase at a magnitude of 2-fold to 4-fold (81). Transcriptome analysis of the *Toxoplasma gondii* tachyzoite cell cycle demonstrated that of the approximately 2,800 genes that are regulated, few undergo changes of large amplitude and about two-thirds of regulated genes undergo changes of less than 4-fold (82) (http://toxodb.org/toxo/). Therefore, giardia and these other protozoan parasites may have evolved such that substantial changes in gene expression at the mRNA level are not required to regulate the cell cycle. However, we examined a small subset of genes in this study so it is possible that genes with greater fold changes during the cell cycle would be identified with a larger-scale study such as one performed by RNA sequencing (RNA-seq) the transcriptome of the elutriation fractions. Furthermore, it would be of interest to examine these genes at the protein level to see if they are regulated posttranscriptionally.

The development of CCE to obtain fractions that can recapitulate the progression of the giardia trophozoites through the cell cycle will allow further studies of this important process in this parasite. These studies have the potential to identify giardia-specific genes and cellular structures that are essential for cell cycle progression that can be used as drug targets to block the proliferation and differentiation of this parasite within a host, limiting the symptoms and spread of giardiasis.

## MATERIALS AND METHODS

### Determination of growth rate and growth conditions for elutriation.

Cultures of *Giardia intestinalis* trophozoites from the WB isolate were grown at 37°C in 16-ml screw-cap glass culture tubes containing modified TYI-S-33 media (83). To determine the cell density of the culture where there was the highest percentage of G_1_/S cells, subsets of the culture tubes were collected at 2.5-h intervals from 10 to 40 h postinoculation, as well as at 1 h and 60 h postinoculation. Cell concentration was determined using an automated cell counter (ViCell XR cell viability analyzer; Beckman-Coulter), and the distribution of cells at different phases of the cell cycle was determined using flow cytometry and analysis performed with FlowJo software (see section below for details).

For each elutriation experiment, a total of 640 ml (40 tubes at 16 ml/tube) of giardia culture was grown to 3 × 10^5^ to 6 × 10^5^ cells/ml. After the tubes were chilled on ice for 5 min and centrifuged (1,100 × *g* for 15 min at 4°C), the pooled cell pellet was resuspended in approximately 2 ml of 1× PBS. An aliquot was removed for cell enumeration. A total of 2 × 10^8^ to 4 × 10^8^ cells were used in each elutriation experiment, although up to 8 × 10^8^ cells in a single experiment have been used. To help visualize the sample, 100 μl of diluted dye stock (the stock was made by adding 2 drops of green food coloring to 2 ml of 1× PBS) was added to the 2-ml giardia sample immediately prior to its injection into the elutriation system. PBS is the buffer used throughout the elutriation process. We have performed elutriations with giardia resuspended in 1× HEPES-buffered saline (HBS) supplemented with 5 mM cysteine and 0.57 mM ascorbic acid to help reduce oxidative stress to the cells. The elutriation of giardia cells in supplemented 1× HBS (data not shown) gave us flow cytometry profiles of the fractions similar to those seen with elutriation performed in unsupplemented 1× PBS. We also tested giardia growth medium (TYI-S-33) as the fluid for elutriation, but this resulted in a poor recovery of cells in the fractions. This was likely due to the increased adhesion of the cells to the tubing during the elutriation process.

### Counterflow centrifugal elutriation.

All elutriation experiments were conducted using a Beckman Coulter, Inc., Avanti J-26 XPI series centrifuge and a JE 5.0 series rotor with the standard 4-ml elutriation chamber. Fluid flow in the system was delivered through silicon tubing (Masterflex catalog no. RK-96420-14) of 1.6-mm inner diameter and was controlled by a peristaltic pump (Masterflex 7523-60 pump drive with a precision flow pump head [catalog no. 77200-60]). The centrifuge rotor was maintained at a speed of 2,400 rpm (550 × *g*) and at a temperature of 21°C. Cells were injected into the elutriation system at a pump flow rate of 1 ml/min.

The flow rates used in elutriation were 5 (F1), 8 (F2), 11 (F3), 14 (F4), 17 (F5), 20 (F6), 25 (F7), 30 (F8), 35 (F9), 40 (F10), 50 (F11), and 60 (F12) ml/min as well as a final blowout (BO) at 60 ml/min with the centrifuge speed adjusted to 0 rpm (see table in Fig. 1A). A total of 50 ml was collected for each fraction, as this corresponded approximately to the dead volume of the system. The cells in each fraction were collected by centrifugation and resuspended in 1 ml of 1× PBS and were then divided into aliquots for cell enumeration, flow cytometry, and RT-qPCR. In addition, the mean size of cells in the unsorted sample prior to elutriation, as well as in samples fractionated by elutriation, was determined using a ViCell XR cell viability analyzer (Beckman-Coulter).

### Flow cytometry.

Giardia trophozoite cells were prepared for flow cytometry following a procedure previously described (11, 14). Briefly, cells were treated with citric acid fixative (40 mM citric acid [monohydrate], 20-mM sodium phosphate [dibasic], 0.2 M sucrose, and 1% Triton X-100) and then diluted in a buffer containing 125 mM MgCl_2_. On the day of flow cytometry, the samples were treated with RNase A and then stained with Sytox green (added to reach a final concentration of 1 μM). Samples were analyzed using a Beckman Coulter, Inc., Cytomics FC 500 instrument. The flow cytometer was calibrated at the beginning of every run using a stained, asynchronous control sample of giardia cells. The flow cytometry data were analyzed using FlowJo analysis software v.7.2.2. Histograms relating the number of cells to the amount of fluorescence per cell were generated.

### Aphidicolin treatment of trophozoites and detection of phosphorylated histone H2AX.

Aphidicolin (Sigma catalog no. A0781) was added to giardia trophozoite cultures to reach a final concentration of 5 μg/ml, and the reaction mixture was incubated at 37°C for 6 h. Control cultures were prepared by incubation of trophozoites with an equivalent volume of dimethyl sulfoxide (DMSO) for 6 h. Following incubation, cells were pelleted by centrifugation and resuspended in 1× PBS.

Preparation of giardia trophozoites for fluorescence microscopy followed a procedure previously described (84). Briefly, trophozoites in 1× PBS were allowed to attach to glass coverslips pretreated with 0.1% polyethylenimine (PEI) under conditions of incubation at 37°C for 10 to 12 min in humidity chambers. The coverslips were then immersed in prechilled methanol at −20°C for 10 min, and the cells were permeabilized with 0.5% Triton X-100. After incubation with blocking buffer (50 mM Tris-HCl [pH 6.8], 150 mM NaCl, 0.5% NP-40, 5 mg/ml bovine serum albumin [BSA]), the coverslips were incubated with a 1:500 dilution of phospho-histone H2AX Ser139 antibody (Active Motif, Inc., catalog no. 39118), washed in 1× PBS, and then incubated with a 1:200 dilution of a fluorescein isothiocyanate (FITC)-conjugated goat anti-rabbit IgG (Jackson ImmunoResearch catalog no. 111-095-003). Cells were postfixed with 3.7% paraformaldehyde, washed with 1× PBS, and then mounted on microscope slides with a drop of Vectashield mounting medium containing DAPI (Vector Laboratories catalog no. H-1200). Coverslips were sealed to the slides with nail polish. Slides were viewed under a Leica DM 6000B epifluorescence microscope. A series of images were taken for each slide using a Leica DFC 350 FX camera and LAS AF v.2.4.1 acquisition software.

A minimum of 200 cells per slide was observed using fluorescent channels for DAPI to visualize the DNA in the nuclei and for FITC to detect the presence of phosphorylated histone H2AX (γH2AX). The number of cells containing positive FITC signal in one or both nuclei was recorded. This procedure was performed for aphidicolin-treated cells, control cells (treated with DMSO alone), and cells from CCE fractions.

### Quantification of DNA content/nuclei in giardia trophozoites.

Giardia cells were attached to polyethylenimine (PEI)-treated coverslips, fixed with methanol, and permeabilized by Triton X-100 as described in the section above. The cells were then incubated with blocking buffer for 1 h, followed by four washes in 1× PBS (5 min per wash). Next, the coverslips were incubated in 3.7% paraformaldehyde for 10 min and were washed twice with 1× PBS (5 min per wash). The coverslips were then incubated in the dark with 0.25 µg/ml of DAPI for 10 min in a lightproof container. The remaining steps were also performed in a light-reduced environment. Coverslips were washed two times in 1× PBS (5 min each) with a final wash in distilled water for 5 min. The coverslips were mounted onto slides using Vectorshield mounting medium without DAPI (Vector Laboratories catalog no. H-1000) and sealed with nail polish. Images were visualized with a Leica DM6000 B epifluorescence microscope. The brightness, gain, intensity, and exposure time were the same for all images taken.

The program CellProfiler (ver. 2.1.1) was used to measure the relative levels of fluorescence intensity of the nuclei. Image files of the DAPI (4,6-diamino-2-phenylindole) staining were uploaded to the software, and modules to identify primary objects (nuclei) and intensity (fluorescence) were selected to measure the objects. For each CCE fraction or unsorted sample, the intensity of each nucleus was recorded for at least 100 cells, and the average intensity and standard deviation were determined.

### Analysis of median bodies in elutriation fractions.

To examine the median bodies, giardia cells from forty 16-ml culture tubes were grown until mid-log phase. Cells were harvested and resuspended in 1× PBS. CCE was performed as described in the previous section. The cells in each fraction were collected by centrifugation, resuspended in 1 ml of 1× PBS, and then divided into aliquots for cell enumeration, flow cytometry, and immunofluorescent microscopy.

Giardia trophozoites were prepared for fluorescence microscopy as previously described (84). The cells on the coverslips were incubated overnight with a 1:75 dilution of the TAT-1 monoclonal antibody (a gift from Keith Gull, University of Oxford), which binds α-tubulin. The cells were washed in 1× PBS and were then incubated with a 1:200 dilution of a Cy3-conjugated goat anti-rabbit IgG (Jackson ImmunoResearch catalog no. 111-165-144). Cells were postfixed with 3.7% paraformaldehyde, washed with 1× PBS, mounted on microscope slides with Vectashield mounting medium containing DAPI (Vector Laboratories catalog no. H-1200), and viewed under an epifluorescence microscope as described in the previous section. A series of images were taken for each slide using a Leica DFC 350 FX camera and LAS AF v.2.4.1 acquisition software. For each elutriation fraction, 50 to 200 cells were examined to determine the percentage of cells that contained a median body (stained by TAT-1). The sizes of the median bodies were calculated by using ImageJ (version 1.47). Briefly, the distance measurement unit of the scale was converted from pixels per inch to pixels per micrometer using the size bar provided by the image acquisition tool and the “Analyze > Set Scale” command. A perimeter was then drawn around each median body with “polygon selections” tool, and the area in square micrometers was obtained using the Measure command in the Analyze menu. The following options were selected in the “Set measurements” check boxes: Area, Min and Max gray values, Perimeter, and Integrated density.

### Reverse transcription-quantitative PCR (RT-qPCR).

Total RNA was extracted from the unsorted asynchronous sample and the CCE fractions using Trizol reagent (Thermo Fisher) according to the manufacturer’s instructions. RNA was converted into cDNA by the use of reverse transcriptase (SuperScript III; Life Technologies, Inc.) from annealed poly(dT)_21_ primers. Each of the 25-μl RT-qPCR mixtures contained 2.5 ng of cDNA and consisted of 1× PCR buffer (200 mM Tris-HCl [pH 8.4], 200 mM KCl, Tween 20, enzyme stabilizers), 2 mM MgCl_2_, 0.4 mM deoxynucleoside triphosphate (dNTP), 8% glycerol, 0.03 μM ROX reference dye, 0.25× SYBR green, 0.25 μM forward and reverse primers, and 0.625 U *Taq* polymerase (BioShop). The PCRs were performed in a Stratagene Mx3000P RT-qPCR instrument with the following parameters: incubation for 4 min at 95°C; incubation for 30 s at 95°C, 60 s at a primer-specific annealing temperature, and 30 s at 72°C, repeated for 40 cycles; and incubation for 60 s at 95°C. The temperature was then raised incrementally from 55 to 95°C to determine the dissociation temperature of the amplified PCR products. All reactions were conducted in 96-well plates sealed with optically clear plastic film (Sarstedt). The sequence of the PCR primers for each gene analyzed is listed in Table 1.

**TABLE 1:** Primers for RT-qPCR

Gene product^a^	ID^b^	Primer direction^c^	Primer sequence (5′ → 3′)
Actin-related protein	GL50803_15113	F	GTCCGTCATACCATCTGTTC
		R	GTTTCCTCCATACCACACG
β-Tubulin	GL50803_101291, GL50803_136020, GL50803_136021	F	GCAGATGCTCAACATCCAGA
		R	TGGAGTTTCCGATGAAGGTC
GAPDH	GL50803_6687	F	GTCTATGAAGCCCGAGGAGA
		R	GGAGCGGAGATGATGACAC
Ribosomal protein L2	GL50803_16086	F	ACAGACAAGCCCCTTCTCAA
		R	GGTCAACAGGGTTCATTGCT
tRNA glycyl synthetase	GL50803_9011	F	GCAAAGCCATTGTTTTACCTCTTC
		R	TGCTATTCCACTACGCCTCA
Ubiquitin	GL50803_7349	F	CGAGAACGCTTCGTGAAATC
		R	GTTTGTGAATAGGCTGTCCGA
Histone H4	GL50803_135001, GL50803_135002, GL50803_135003	F	GTGGAGGTGTGAAGCG
		R	TGTGTATGTGAGGGAGTCG
Histone H3	GL50803_14212, GL50803_35231	F	TACCAGAAGTCCACAGACC
		R	TGGAAGCGGATGTCGGA
Histone H2A	GL50803_14256, GL50803_27521	F	GTCGTGGCAGAGGTCTT
		R	CTCCTTGTCCTTGCGGA
Histone H2B	GL50803_121045, GL50803_121046	F	GACAACATCCGCTCCGA
		R	CGAAGAGGTCGTTCACG
Cyclin A	GL50803_14488	F	ACGGCAGCAGGCGTTTTA
		R	CAGCATCTCTGGACTGTAAGT
Cyclin B	GL50803_3977	F	TAGACGACGAATCCCAGA
		R	GAATGTCCGTTTCTTTATCG
Cyclin B-like	GL50803_17505	F	GAAAACAGAGATACGCCTGCT
		R	GCAAAATACGCACAAATAGCCG
MCM5	GL50803_89112	F	CCTCGGTCGTTTTGATGACTT
		R	GTCTCTTTCTGGGCTCTGTTT
PCNA	GL50803_6564	F	GATTCGGGTCAAAAATGAGGTC
		R	GGCGAGTTATCACTGAGTTC
PLK	GL50803_104150	F	TCTCTCCCAACGCTCTCAT
		R	AGAATGGTAGTCCCCTCTTC
Thymidine kinase	GL50803_8364	F	TTCAGTCAGAGTCGGAGGA
		R	AGACAAGGACGATAGAGTGC

a The products of the six genes evaluated as candidate references or normalizers for RT-qPCR are listed first, followed by the products of the 11 genes evaluated for their cell cycle-regulated expression.

b The gene identifiers (ID) are from GiardiaDB (http://giardiadb.org/giardiadb/).

c "F" indicates the forward direction of the PCR primer; "R" indicates the reverse direction.

Three replicates of each cDNA sample were analyzed in each RT-qPCR run, along with two control samples. The first control sample was the NoRT (no reverse transcriptase) control, which consisted of 2.5 ng RNA in the place of cDNA to test for genomic DNA contamination. The second control sample was the NTC (no-template control), which consisted only of reaction mix and diethyl pyrocarbonate (DEPC)-H_2_O to test for the presence of reagent contamination and the formation of primer dimers. In addition, standards containing 20, 10, 5, 2, and 1 ng cDNA were prepared by serial dilution and analyzed to determine the levels of efficiency for each primer pair.

The relative expression levels of the suspected cell cycle genes were determined using the threshold cycle (2^−ΔΔ*CT*^) method with the actin-related gene serving as the normalizer. Fraction 5 was chosen as the calibrator for this analysis, as that was the fraction that was most concentrated in a single-cell cycle phase (G_2_) and consistent in DNA content across the experiments. Two independent elutriation experiments, or biological replicates, with four independent cDNA syntheses each were analyzed using RT-qPCR. Differences among fraction means were tested using one-way analysis of variance (ANOVA). The results showed significant differences among the fractions at *P* = 0.03.

### Analysis of reference genes for RT-qPCR with geNorm.

The geNorm VBA (Visual Basic for Applications) applet for Microsoft Excel was kindly donated by Jo Vandesompele from the Center for Medical Genetics in Belgium. Quantitative PCR was performed with elutriation fractions and primers for six potential normalizer genes encoding the following products: actin-related protein, β-tubulin, glyceraldehyde-3-phosphate dehydrogenase (GAPDH), glycyl tRNA synthetase, ribosomal protein L2, and ubiquitin. Following RT-qPCR, the relative quantities, or nonnormalized expression values (*Q*), of each sample for each primer pair were determined using the following formula:

$$Q=E^{\left(\text{min}\;Cq\;-\;\text{sample}\;Cq\right)}$$

where *E* is the amplification efficiency for each primer pair, adjusted such that *E* = 2 when the efficiency is 100%, and min *C_q_* is the lowest quantification cycle (*C*_*q*_) value, or the *C_q_* value of the sample with the highest expression. These values were entered into the geNorm program, which then calculated gene-stability measures (M values) for each suspected reference gene. This analysis was conducted for two independent elutriation experiments, and the genes with the lowest M values were considered the most suitable for use in giardia cell cycle analysis.
